# Variations in Root Characteristics and Cadmium Accumulation of Different Rice Varieties under Dry Cultivation Conditions

**DOI:** 10.3390/plants13172457

**Published:** 2024-09-02

**Authors:** Chaoping Shan, Can Shi, Xinran Liang, Yanqun Zu, Jixiu Wang, Bo Li, Jianjun Chen

**Affiliations:** College of Resources and Environment, Yunnan Agricultural University, Kunming 650201, China; shanchaoping05@163.com (C.S.);

**Keywords:** rice under dry cultivation, cadmium, genotypic differences, root characteristics

## Abstract

Variations in the cadmium (Cd) accumulation and root characteristics of different genotypes of rice during three developmental periods of dry cultivation were investigated in pot experiments in which two levels of Cd were added to the soil (0 and 10 mg kg^−1^). The results show that the Cd concentration in each organ of the different rice genotypes decreased in both the order of roots > shoots > grains and during the three developmental periods in the order of the maturity stage > booting stage > tillering stage. The lowest bioaccumulation factor (BCF) and translocation factor (TF) were found in Yunjing37 (YJ37) under Cd stress. At maturity, Cd stress inhibited the root length of Dianheyou34 (DHY34) the most and that of Dianheyou 918 (DHY918) the least, also affecting the root volume of DHY34 and Dianheyou615 (DHY615) the most and that of YJ37 and Yiyou 673 (YY673) the least; the inhibition rates were 41.80, 5.09, 40.95, and 10.51%, respectively. The exodermis showed the greatest thickening in YY673 and the lowest thickening in DHY615, while the endodermis showed the opposite result. The rates of change were 16.48, 2.45, 5.10, and 8.49%, respectively. The stele diameter of DHY615 decreased the most, and that of YY673 decreased the least, while the secondary xylem area showed the opposite result; the rates of change were −21.50, −14.29, −5.86, and −26.35%, respectively. Under Cd stress treatment at maturity, iron plaque was extracted using the dithionite–citrate–bicarbonate (DCB) method. The concentration of iron (DCB-Fe) was highest in YJ37, and the concentration of cadmium (DCB-Cd) was lowest in DHY34. YJ37 was screened as a low Cd-accumulating variety. The concentration of available Cd in the rhizosphere soil, iron plaque, root morphology, and anatomy affect Cd accumulation in rice with genotypic differences. Our screening of Cd-accumulating rice varieties provides a basis for the dry cultivation of rice in areas with high background values of Cd in order to avoid the health risks of Cd intake.

## 1. Introduction

Research on the contamination of arable soil by heavy metals over the past 10 years has shown that 80% of urban soils in China are contaminated by cadmium (Cd) [1]. Cd contamination of arable land in Southwest China is caused by a high geochemical background [2], secondary enrichment during soil formation, and human activities. Cd possesses strong chemical activity in soil [3] and is a non-essential element for human and plant growth. Cd prevents plants from absorbing nutrients and inhibits plant growth and development. The question of how to control Cd uptake by plants is an important environmental problem that needs to be solved.

Rice (*Oryza sativa* L. var. *formosana* ) is both a major food crop and a large consumer of water; it is consumed by more than 65% of the population of China as a staple food [4]. The water consumption of rice irrigation accounts for about 65% of the total water consumption in irrigated agriculture [5]. China is 1 of the 13 arid countries in the world [6], and water scarcity has become the main reason affecting rice production. Although the southern rice production area in China experiences abundant rainfall, it is affected by the monsoon climate, and seasonal drought often occurs [7], resulting in very limited water resources for growing rice. With the “rice–water” contradiction becoming increasingly prominent, technology for dry rice cultivation has gradually developed. Dry cultivation does not require seedlings or transplants; the rice is sown directly in dryland conditions. This method mainly depends on natural precipitation throughout the reproductive period, with appropriate recharge only performed during critical periods of water demand or drought, and no water layer is used during the entire growth period [8], which can save 50% of water resources compared to ordinary rice cultivation [9]. In 2023, Yunnan Province promoted a dry rice cultivation area of 6.67 × 10^4^ hm^2^, achieving additional rice production of 3 × 10^5^ t [10]. The dry cultivation of rice provides an opportunity to increase productivity; however, the technique is hampered by limitations in terms of rice yield and quality [8]. Deng et al. [11] showed that the dry cultivation of rice increases Cd concentration in grains. Methods of improving yield and quality and ensuring the safe production of rice under dry cultivation pose scientific issues.

Rice is a monocotyledonous plant that readily absorbs Cd [12]. Most of the Cd^2+^ absorbed by rice accumulates in the roots, causing severe inhibition of root growth, bending, and reductions in the number of root tips [13]. The Cd^2+^ absorption of rice mainly depends on its genetic factors and external environmental conditions. In terms of genetic factors, there are significant genotypic differences in the uptake of heavy metals by crops. Feng et al. [14] showed that Cd uptake and accumulation in various organs of rice plants varied greatly among genotypes. In terms of external environmental conditions, Chi et al. [15] showed that the environment contributed most to the variation in grain Cd concentration, accounting for 87% of the total variation. The main difference in the growing environment under dry rice cultivation conditions relative to traditional flooded rice is the substantially lower moisture. Changes in water conditions affect the formation of iron plaque on the surface of rice roots, the soil potential of hydrogen (pH) [16], and the electric potential half cells (Eh) [17], thereby affecting the forms and effective concentration of soil Cd and the uptake and translocation of Cd in rice [18].

At present, studies on Cd uptake and accumulation in rice under dry cultivation conditions have mainly used upland rice to discuss the effects of Cd uptake and accumulation. The effects of water management (flooding, alternate wetting and drying, and dry farming) on Cd accumulation in rice have been studied, but few studies have been conducted on Cd uptake and accumulation in rice under dry cultivation conditions. In this study, it was hypothesized that Cd treatment would have a significant effect on the uptake of accumulated Cd in rice under dry cultivation conditions and that there would be genotypic differences; the genotypes DHY34, DHY615, DHY918, YY673, and YJ37 were used as research materials to explore these genotypic differences and the characteristics of root accumulation. Our screening of rice varieties that show low Cd accumulation provides theoretical support for the safe production of rice using dry cultivation in areas with high background values of Cd.

## 2. Results

### 2.1. Effects of Cd Stress on Cd Accumulation in Different Genotypes of Rice under Dry Cultivation Conditions

#### 2.1.1. Effects of Cd Stress Treatments on Cd Concentrations in Various Rice Organs and Rhizosphere Soil under Dry Cultivation Conditions

**Figure 1:**
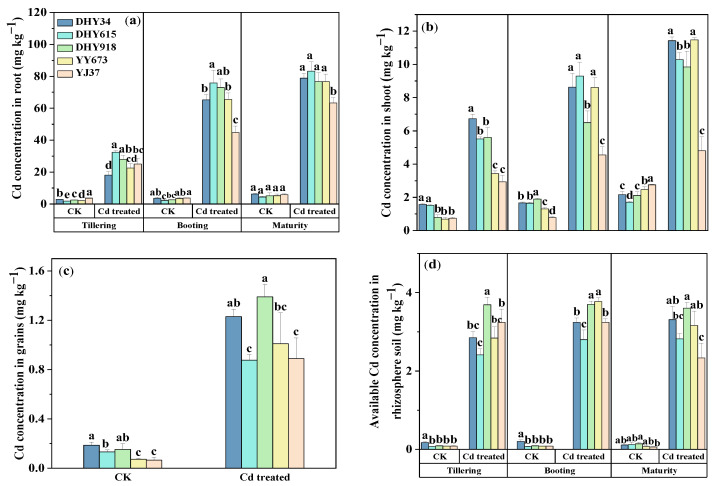
The Cd concentrations in the various organs and rhizosphere soil of different genotypes of rice under dry cultivation conditions. (**a**) Cd concentrations in roots of different genotypes of rice in three developmental periods (*n* = 3); (**b**) Cd concentrations in shoots of different genotypes of rice in three developmental periods (*n* = 3); (**c**) Cd concentrations in grains of different genotypes of rice at maturity stage (*n* = 3); (**d**) available Cd concentrations in the rhizosphere soil of different genotypes of rice in three developmental periods. The values are the mean ± standard deviation (*n* = 3). Different letters represent significance at *p* < 0.05 (Duncan) among the different genotypes of rice under the same treatment during the same stage of dry cultivation. CK represents a concentration of 0 mg kg^−1^ Cd added externally to the soil. Cd treatment represents a concentration of 10 mg kg^−1^ Cd added externally to the soil. Abbreviations: DHY34, Dianheyou 34; DHY615, Dianheyou 615; DHY918, Dianheyou 918; YY673, Yiyou 673; YJ37, Yunjing 37.

The Cd concentrations in rice organs and the available Cd in rhizosphere soil under the Cd stress treatment were higher than when Cd was added externally to the soil at 0 mg kg^−1^ (CK). In the three developmental periods of both treatments, the root Cd concentrations were much higher than the shoot Cd concentrations (Figure 1b), and the shoot Cd concentrations were much higher than the grain Cd concentrations (Figure 1c).

As shown in Figure 1a, the different genotypes showed significant differences (*p* < 0.05) in root Cd concentrations under the two treatments. Under the CK treatment, the highest root Cd concentration was found in YJ37, while DHY615 showed the lowest concentration at both the tillering stage and the booting stage. Under the Cd stress treatment, the highest Cd concentrations were found in the roots of DHY615 and DHY918 in three developmental periods. The lowest Cd concentrations were found in the roots of DHY34 at the tillering stage and in the roots of YJ37 at the booting and maturity stages. The DHY615 had the lowest root Cd concentration under the CK treatment and the highest root Cd content under the Cd stress treatment, indicating that more Cd was absorbed by DHY615 roots under high Cd stress. The average root concentrations of Cd in the three developmental periods under Cd stress conditions were 25.17, 64.89, and 75.72 mg kg^−1^, and the coefficients of variation were 20.94, 17.97, and 10.13%, respectively, showing a decreasing trend from the maturity stage > booting stage > tillering stage.

As shown in Figure 1b, the different genotypes showed significant differences (*p* < 0.05) in shoot Cd concentrations under the two treatments, which increased as the concentration of Cd increased. The shoot Cd concentration in DHY34 was the highest in the three developmental periods of the Cd stress treatment, while the shoot Cd concentration in YJ37 was the lowest. Genotypic differences in the ability of rice roots to transport shoot Cd were shown. The average shoot Cd concentrations in the three developmental periods of Cd stress treatment were 4.84, 7.52, and 9.57 mg kg^−1^, with coefficients of variation of 30.17, 24.62, and 26.32%, respectively, which also showed a decreasing trend from the maturity stage > booting stage > tillering stage.

As shown in Figure 1c, the different genotypes showed significant differences (*p* < 0.05) in grain Cd concentrations under the two treatments. The Cd concentration of the rice grains of all genotypes of CK treatment did not exceed the standard (GB 2762-2022 [19], 0.2 mg kg^−1^), decreasing from DHY34 > DHY918 > DHY615 > YY673 > YJ37. Under the Cd stress treatment, the grain Cd concentrations exceeded the standard. The order of Cd concentration in the rice grains was DHY918 > DHY34 > YY673 > DHY615 > YJ37. This indicates that DHY34 and DHY918 grains are more capable of Cd bioaccumulation, while YJ37 grains are less capable. The lowest Cd concentration was found in YJ37 under both treatments.

As shown in Figure 1d, there were significant differences (*p* < 0.05) in the available Cd concentrations in the rhizosphere soil of the different rice genotypes. In the three developmental periods of the CK treatment, the highest available Cd concentrations in rhizosphere soil were found in DHY34, while the lowest was found in YJ37. Under the Cd stress treatment, the highest available Cd concentration in the rhizosphere soil of the three developmental periods was found in DHY918, and the lowest was found in DHY615. The mean available rhizosphere soil Cd concentrations in the three developmental periods were 3.01, 3.35, and 3.04 mg kg^−1^, and the coefficients of variation were 15.75, 11.06, and 16.39%, respectively, showing a decreasing trend from the booting stage > maturity stage > tillering stage.

#### 2.1.2. Effects of Cd Stress Treatments on Rice BCF and TF under Dry Cultivation Conditions

**Figure 2:**
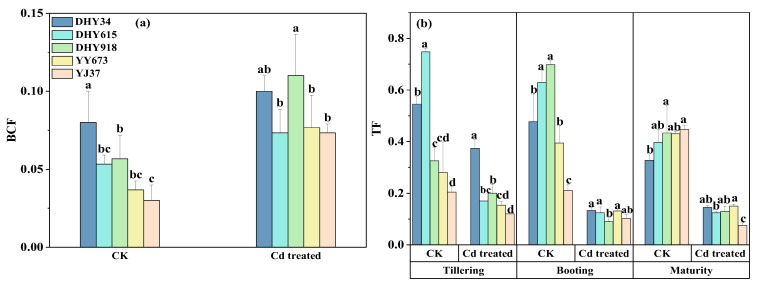
The effect of Cd stress treatments on the BCF (**a**) and TF (**b**) values of different genotypes of rice under dry cultivation conditions. The BCF values use the date at maturity, and the TF values use the date in three developmental periods. The BCF was calculated using Equation (1) to evaluate the accumulation of Cd in soils and grains. The TF was calculated using Equation (2) to evaluate the upward conduction ability of Cd in roots and shoots. The values are the mean ± standard deviation (*n* = 3). Different letters represent significance at *p* < 0.05 (Duncan) among the different genotypes of rice under the same treatment during the same stage of dry cultivation. CK represents a concentration of 0 mg kg^−1^ Cd added externally to the soil. Cd treatment represents a concentration of 10 mg kg^−1^ Cd added externally to the soil. Abbreviations: DHY34, Dianheyou 34; DHY615, Dianheyou 615; DHY918, Dianheyou 918; YY673, Yiyou 673; YJ37, Yunjing 37.

As shown in Figure 2a, there were significant differences (*p* < 0.05) in the BCFs of the different rice genotypes under the two treatments. The BCF values of the five rice genotypes under the Cd stress treatment were higher than those in the CK treatment. Under Cd stress treatments, the BCFs decreased in the order of DHY918 > DHY34 > YY673 > DHY615 > YJ37, which was consistent with the Cd concentration in the grains. This indicates that the differences between rice varieties have a significant effect on enrichment capacity, with YJ37 having a lower Cd bioaccumulation capacity. The variation range of the BCF values of the five rice grains under the CK treatment was 0.02~0.10, with a coefficient of variation of 40.42%. Under the Cd stress treatment, the range in variation of the BCF values of the five rice grains was 0.06~0.14, with a coefficient of variation of 23.51%.

As shown in Figure 2b, there were significant differences (*p* < 0.05) in the Cd TF values of the different genotypes of rice under the two treatments. The TF values of the five rice varieties in the three developmental periods under the Cd stress treatment were lower than those of the CK treatment. The TF of DHY34 in the three developmental periods under the Cd stress treatment was larger, while that of YJ37 was smaller. This indicates that there are differences in the Cd translocation capacity of different rice varieties. Under the Cd stress treatment, the TF values in the three developmental periods showed ranges of variation from 0.12 to 0.41, 0.07 to 0.15, and 0.08 to 0.16, with coefficients of variation of 45.03, 18.48, and 22.58%, respectively. The TF values at the three developmental periods decreased from the booting stage > tillering stage > maturity stage under the Cd stress treatment.

#### 2.1.3. Cluster Analyses Based on Cd Cumulative Trait Phenotypes

**Figure 3:**
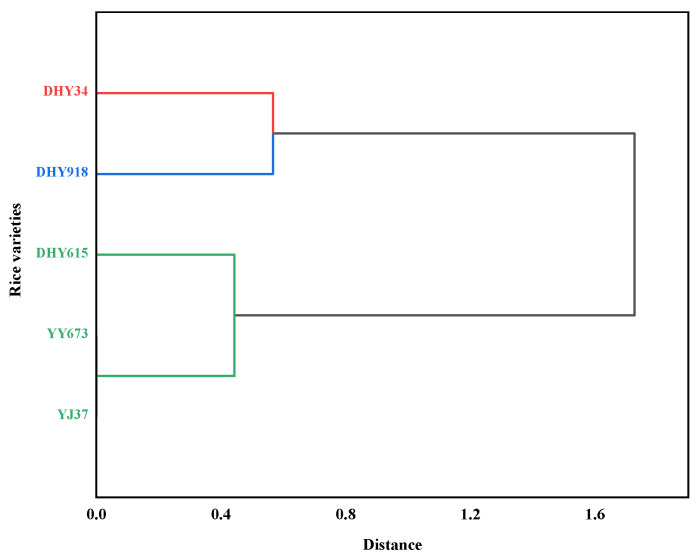
Cluster analyses of Cd accumulation characteristic phenotypes. The grain Cd concentration, Cd accumulation, BCFs, and TFs of five different genotypes with Cd stress under dry cultivation conditions (*n* = 3). The Cd concentrations in grains and BCFs use the date at the maturity stage, while the Cd accumulation and TFs use the dates for the three developmental periods. Abbreviations: DHY34, Dianheyou 34; DHY615, Dianheyou 615; DHY918, Dianheyou 918; YY673, Yiyou 673; YJ37, Yunjing 37.

Cluster analyses of the grain Cd concentrations, Cd accumulation, BCFs, and TFs of the five different genotypes treated with Cd stress under dry cultivation conditions were carried out, and the results are shown in Figure 3. DHY34 was classified into one category, DHY918 into a second category, and DHY615, YY673, and YJ37 into a third category. YJ37 was found to be a grain variety with low Cd accumulation.

### 2.2. Characteristics of Different Genotypes’ Root Cd Accumulation with Cd Stress Treatments under Dry Cultivation Conditions

#### 2.2.1. The Effects of Cd Stress Treatments on the Root Morphology of Different Genotypes of Rice under Dry Cultivation Conditions

**Figure 4:**
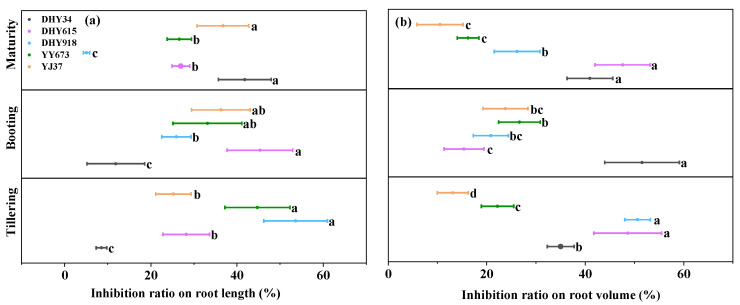
The effect of the Cd stress treatment on the root morphology of different genotypes of rice under dry cultivation. (**a**) Inhibition rate of Cd stress treatment on the root lengths of 5 genotypes of rice under dry cultivation conditions in three developmental periods. (**b**) Inhibition rate of Cd stress treatment on the root volume of 5 genotypes of rice in three developmental periods. Fresh and clean whole roots were used to analyze root morphology. The inhibition rate was obtained using Equation (3) and represents the degree of inhibition of rice root morphology under the Cd stress treatments. The values are the mean ± standard deviation (*n* = 3). Different letters indicate significance at *p* < 0.05 (Duncan) between the inhibition rates of the Cd stress treatments on the root length and root volume of different genotypes of rice at the same stage under dry cultivation conditions. Abbreviations: DHY34, Dianheyou 34; DHY615, Dianheyou 615; DHY918, Dianheyou 918; YY673, Yiyou 673; YJ37, Yunjing 37.

As shown in Figure 4a, the three developmental periods of the Cd stress treatment had inhibitory effects on the rice’s root length and volume. At the tillering stage, DHY918 showed the highest inhibition of root length, while DHY34 showed the least; at the booting stage, DHY615 showed the highest inhibition of root length, and DHY34 showed the least; and at the maturity stage, DHY34 showed the highest inhibition of root length, and DHY918 showed the least. The average decreases in root length caused by Cd stress in the three developmental periods were 32.07, 30.51, and 27.44%, with coefficients of variation of 54.87, 41.08, and 51.31%, respectively, showing a decreasing trend from the tillering > maturity > booting stages.

As shown in Figure 4b, at the tillering stage, DHY918 and YJ37 showed the greatest and lowest inhibition of root volume by Cd stress, respectively. At the booting stage, DHY34 and DHY615 showed the greatest and lowest inhibition of root volume by Cd stress, respectively. At maturity, Cd stress inhibited the root volume the most in DHY615 and the least in YJ37. The average decreases in root volume by Cd stress at the three developmental periods were 33.91, 27.64, and 29.23%, and the coefficients of variation were 48.18, 50.62, and 61.54%, respectively. The inhibition rates of Cd stress on root volume at the three developmental periods decreased from the tillering stage > maturity stage > booting stage, which is consistent with the root length result.

#### 2.2.2. The Effects of Cd Stress on the Anatomic Structure of Roots of Different Rice Genotypes at Maturity under Dry Cultivation Conditions

**Figure 5:**
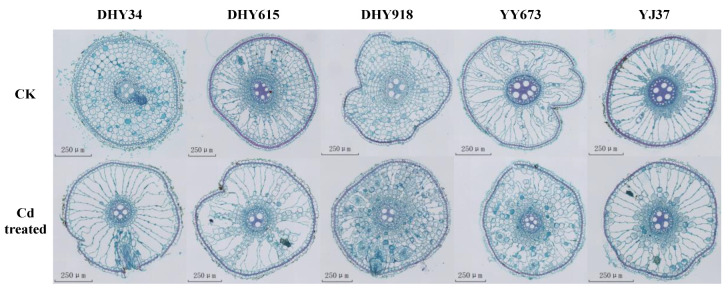
The anatomy of the root systems of different rice genotypes under dry cultivation conditions. The scale bar is 250 μm. The number of rice root sections used for anatomical observation was 30 (*n* = 3). Abbreviations: DHY34, Dianheyou 34; DHY615, Dianheyou 615; DHY918, Dianheyou 918; YY673, Yiyou 673; YJ37, Yunjing 37.

The barrier structure of plants consists of the suberin and lignification endodermis and exodermis of the Casparian strip. As shown in Figure 5, the root system of the CK-treated rice showed an intact morphology and structure. The Cd stress treatment significantly affected the structure of the rice root system, increasing the thickness and deformity of the exodermis and the endodermis. The stability of rice roots under Cd stress was reduced, and they became more susceptible to toxicity.

**Figure 6:**
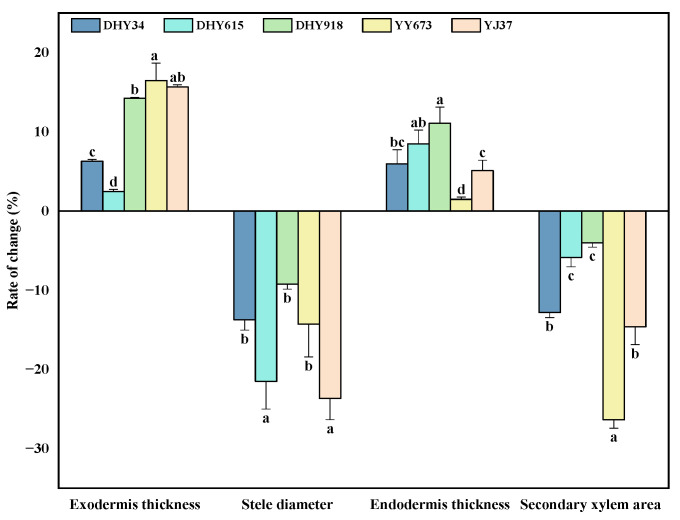
Rates of change in the anatomical parameters of the root systems of different rice genotypes under dry cultivation conditions. The rates of change were calculated using Equation (3) to represent the extent of the effect of the Cd stress treatment on rice. The values are the mean ± standard deviation (n = 3). Different letters indicate significance at p < 0.05 (Duncan) among the change rates. Abbreviations: DHY34, Dianheyou 34; DHY615, Dianheyou 615; DHY918, Dianheyou 918; YY673, Yiyou 673; YJ37, Yunjing 37.

The rates of change in the anatomical parameters of the roots of the different rice genotypes at maturity are shown in Figure 6. Cd stress increased the thickness of the exodermis and endodermis and decreased the secondary xylem area and the stele diameter in all five rice varieties; this treatment had a greater effect on the exodermis of YY673 and YJ37 and a smaller effect on that of DHY615. The Cd stress treatment had a greater impact on the stele diameter of YJ37 and DHY615 and a smaller impact on that of DHY918, DHY34, and YY673. The Cd stress treatment had a greater impact on the endodermis of DHY918 and DHY615 and a smaller impact on that of YY673. The Cd stress treatment had a greater impact on the area of the secondary xylem of YY673 and a smaller impact on that of DHY918 and DHY615, which is the inverse of the results for the endodermis. Compared to the CK treatment, the Cd stress treatment increased the exodermis and endodermis thicknesses by averages of 10.79 and 6.08%, respectively; the coefficients of variation were 53.79% and 54.48%. The stele diameter and secondary xylem area were decreased by averages of 16.48% and 11.76%, respectively; the coefficients of variation were 35.10% and 62.34%, respectively.

#### 2.2.3. The Effects of Cd Stress Treatments on the DCB-Fe and DCB-Cd Concentrations of Different Rice Genotypes under Dry Cultivation Conditions

**Figure 7:**
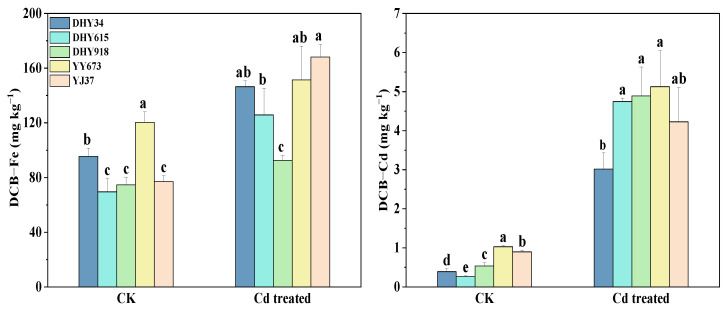
Effects of Cd stress treatments on DCB-Fe and DCB-Cd concentrations in different rice genotypes at maturity under dry cultivation. DCB-Fe indicates the Fe concentration in the iron plaque on the root surface, and DCB-Cd indicates the Cd concentration in the iron plaque on the root surface. The values are the mean ± standard deviation (*n* = 3). Different letters represent the variability of DCB-Fe and DCB-Cd concentrations among different rice genotypes in the same treatment at maturity. Abbreviations: DHY34, Dianheyou 34; DHY615, Dianheyou 615; DHY918, Dianheyou 918; YY673, Yiyou 673; YJ37, Yunjing 37.

As shown in Figure 7, there were significant differences (*p* < 0.05) in the DCB-Fe and DCB-Cd concentrations in the different rice genotypes at maturity. The DCB-Fe and DCB-Cd concentrations increased with increasing Cd treatment concentrations. Under the Cd stress treatment, the DCB-Fe concentration was the highest in YJ37 and the lowest in DHY918. DHY615, DHY918, and YY673 had higher DCB-Cd concentrations, while DHY34 had the lowest DCB-Cd concentration. This indicates that DHY615, DHY918, and YY673 trapped more Cd in the iron plaque on the root surface, facilitating a reduction in Cd uptake by rice roots.

### 2.3. Correlation Analysis Based on the Cd Accumulation Characteristics of Rice at Maturity

**Figure 8:**
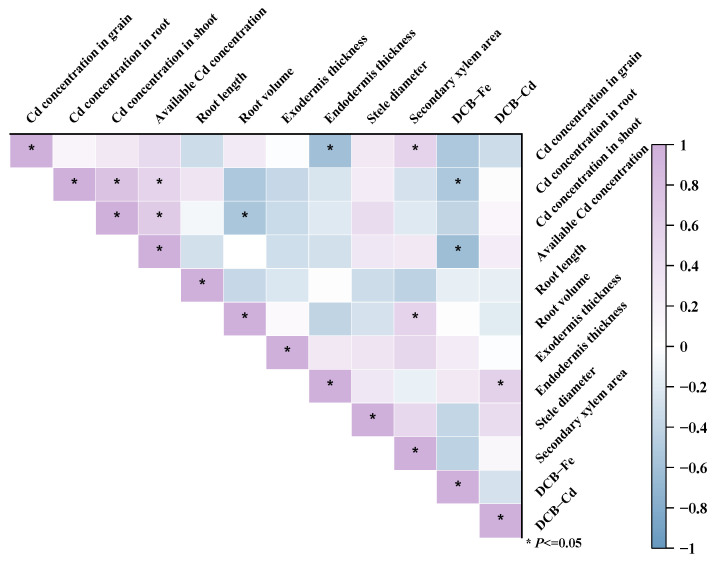
Correlation analysis of Cd accumulation in different genotypes of rice at maturity under dry cultivation. Correlation analyses (Pearson, *p* ≤ 0.05) between Cd concentrations in various rice organs and available Cd concentrations in the rhizosphere soil, root morphology, root anatomy, DCB-Fe, and DCB-Cd at maturity under Cd stress treatments. Asterisks indicate significance at * *p* ≤ 0.05.

The correlations between the grain Cd concentration and the other indexes of rice under the Cd stress treatment at maturity are shown in Figure 8. There was a negative correlation between the grain Cd concentration and endodermis thickness but a positive correlation with the secondary xylem area. The root Cd concentrations were positively correlated with both the shoot Cd concentrations and the concentrations of available Cd in the rhizosphere soil and negatively correlated with the DCB-Fe concentrations. The shoot Cd concentrations were positively correlated with the Cd concentrations in the rhizosphere soil and the root Cd concentrations. The endodermis thickness and DCB-Cd were positively correlated.

**Figure 9:**
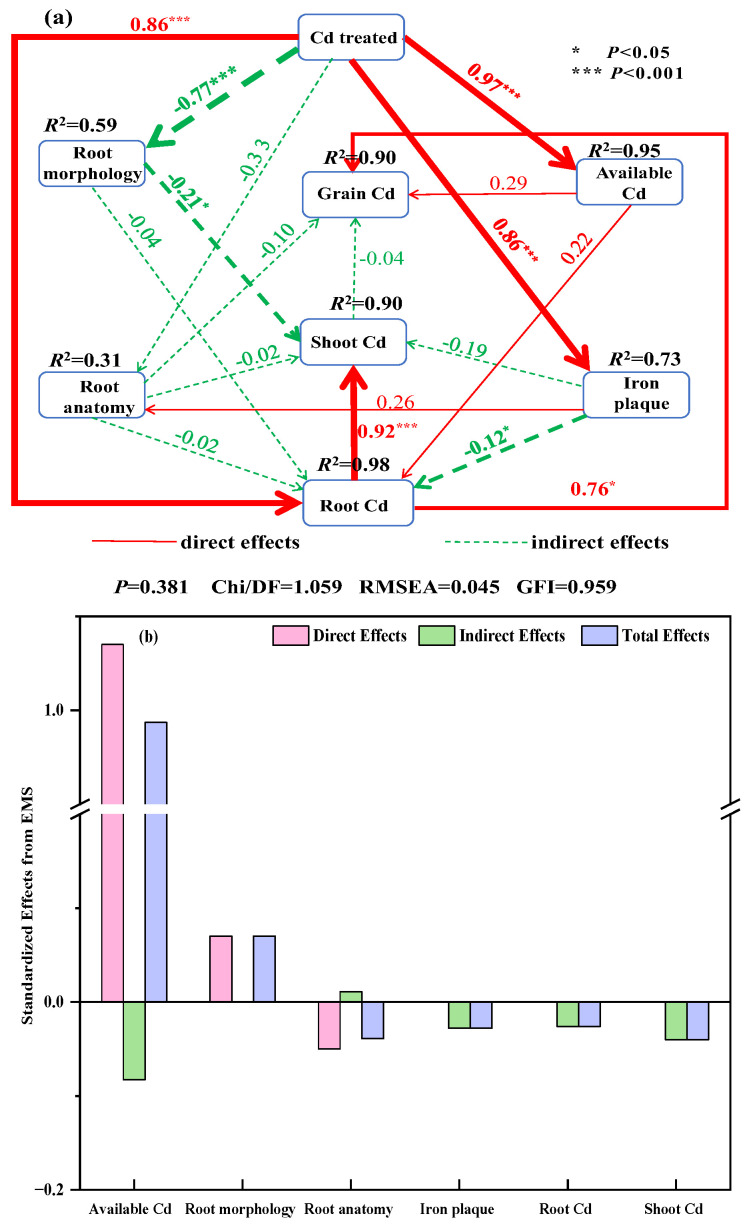
An SEM showing the direct and indirect effects of Cd treatment, the available Cd concentrations in the rhizosphere soil, root morphology, root anatomy, root surface iron plaques, root Cd concentration, and shoot Cd concentration on grain Cd concentration (**a**). Standardized direct, indirect, and total effects of Cd treatment, available Cd concentrations in the rhizosphere soil, root morphology, root anatomy, root surface iron plaques, root Cd concentration, and shoot Cd (**b**). An SEM plotted using maturity data. Root morphology was analyzed using principal components for root length and root volume, and the first principal component was selected with a contribution of 78.60%. Root anatomy was analyzed using principal components for exodermis and endodermis thickness, and the first principal component was selected with a contribution of 71.13%. Iron plaque was analyzed using principal components for DCB-Fe and DCB-Cd concentration, and the first principal component was selected with a contribution of 81.73%. Numbers adjacent to arrows are standardized path coefficients, analogous to relative regression weights, and are indicative of the magnitude of the correlation. Continuous and dashed arrows indicate positive and negative relationships, respectively. The arrow width is proportional to the strength of the relationship. The proportion of variance explained (*R*^2^) appears alongside every response variable in the model. Goodness-of-fit statistics for the model are shown at the bottom of the image. * *p* < 0.05; *** *p* < 0.001.

To clarify the effect and relative importance of Cd treatment on grain Cd concentrations in rice, a structural equation model (SEM) was generated based on the known effects and relationships among Cd treatments, available Cd concentrations in the rhizosphere soil, root morphology, root anatomy, root surface iron plaques, root Cd concentrations, shoot Cd concentrations, and grain Cd concentrations (Figure 9a). The fitted models explain 90% of the variance in rice Cd. The Cd treatments had significant direct positive effects on the available Cd concentrations in the rhizosphere soil, root surface iron plaques, and root Cd concentration and had direct negative effects on the root morphology. The root Cd concentration had a significant direct positive effect on the shoot Cd concentration and grain Cd concentration. The root surface iron plaques had a direct negative effect on the root Cd concentration. The root morphology had a direct negative effect on shoot Cd. In addition, the available Cd concentration in the rhizosphere soil was the most important indicator of rice grain Cd, showing the highest standardized total effect (direct plus indirect effects reached 0.95), followed by root morphology, root Cd concentration, root surface iron plaque, root anatomy, and shoot Cd concentration (Figure 9b).

## 3. Discussion

Genotypic differences were found in rice regarding Cd accumulation, and the Cd concentration in the studied rice grains was highly variable, with a maximum difference of 47.0% among the varieties [20]. The average reduction in rice shoot Cd concentrations was 42.26% under flooded conditions compared to non-flooded conditions [21]. The Cd concentrations in the roots, shoots, and grains of YJ37 under Cd stress were lower under dry cultivation (Figure 1). The Cd concentrations in the rice grains of the five different genotypes were compared with the national standard of 0.2 mg kg^−1^ GB 2762-2022 [19]. The Cd concentrations in rice grains under the CK treatment did not exceed the standard, and the Cd concentrations in rice grains under Cd stress exceeded the standard in all cases. The distribution of Cd concentrations in each rice organ was in the order of roots > shoots > grains; the distribution and accumulation of Cd in the different organs of rice was tissue-specific [22]. Huang et al. [23] demonstrated that 98% of Cd in rice is absor))bed through the root system during the grain filling stage. Cd uptake by roots dominated Cd accumulation in rice plants [24]. The Cd concentrations in the roots and shoots of the rice in the three developmental periods showed a decreasing order from maturity > booting > tillering, which increased with the prolongation of fertility (Figure 1); this is in line with the findings of other researchers [25]. The crop uptake of heavy metals in external environmental conditions is also an important factor. Compared to flooding, alternate wetting and drying conditions [26] and dry farming [27] increased the concentrations of available Cd in the soil, which promoted Cd translocation and accumulation in plants. The available Cd concentrations in the rhizosphere soil were positively correlated with root and shoot Cd concentrations (Figure 8). Changes in soil Eh and pH under dry cultivation may increase the availability of Cd and increase its uptake by rice [28].

The shoot Cd concentration was positively correlated with the root Cd concentration in the five genotypes under dry cultivation conditions (Figure 8). The shoot Cd concentration was influenced by the uptake and translocation of Cd in the roots. The TF values indicate the possibility of Cd translocation and distribution in different rice tissues [29]. The lowest TF values of YJ37 occurred in three developmental periods under the Cd stress treatment (Figure 2b), which might be related to the expression of *OsNramp5*, *OsNramp1*, *OsIRTs*, and *OsHMA2*, the main Cd transport proteins in the rice root system; the overexpression of these proteins favors the transport of Cd from the roots to the shoots [30]. The transport of Cd from the roots to the shoots could explain the differences in the accumulation of Cd in the shoots of the different genotypes of rice studied. The TF values in the different rice fertility stages decreased from booting > tillering > maturity (Figure 2b). The root translocation coefficients of different rice varieties increased with the prolongation of the fertility period and reached a maximum at the booting or full heading stage [31].

Cd stress decreased the total root length, root volume, mean root diameter, and root activity in rice [32]. It also inhibited root length and root volume in the rice under dry cultivation conditions with genotypic differences (Figure 4). Liu et al. [33] found that compared to high-Cd cultivars, low-Cd cultivars possess stronger root systems characterized by a greater surface area, root length, and volume, but a lower apical surface area, under high Cd stress. The uptake of Cd by rice mainly depends on the apical surface area and the number of root tips [13]. The larger the apical surface area and the greater the number of root tips, the greater the amount Cd absorbed. Cd competes with major rice nutrients, transfers to shoot organs, and causes a decline in growth. Changes in root morphology affect Cd uptake in rice [34]. The effect of Cd on root morphology depends on the rice variety [35].

In the process of rice Cd uptake and transport, the anatomy of the roots is affected by genotype [36] and cultivation conditions [37]. The exodermis and endodermis of the roots of different genotypes of rice thickened under Cd stress (Figure 6). This indicates that rice has a higher tolerance to Cd and a greater ability to prevent the apoplastic movement of Cd [38]; this, in turn, impedes Cd from entering the stele, effectively limiting the transport of Cd [39]. YY673 showed the greatest thickening of the exodermis, while DHY615 showed the least, and the changes in endodermis thickening showed an inverse trend. In addition, the highest decrease in the stele diameter was shown in DHY615, while the smallest decrease was shown in YY673, and the inverse trend was shown for the secondary xylem area (Figure 6). Possibly due to the earlier formation of barriers in DHY615 and YY673, reducing bypass flow more efficiently, more Cd was retained in the root during apoplastic translocation [40]. The root Cd concentration of YY673 was the highest in all three developmental periods (Figure 1). The negative correlation between the Cd concentration in grains and endodermis thickness (Figure 8) may be because Cd stimulates the deposition of more suberin and lignin in the endodermis and exodermis of rice roots, which forms more mature casparian and restricts the transport of Cd to the xylem [13]. The Cd concentration in the grains and secondary xylem area were positively correlated (Figure 8). An increase in the secondary xylem area may lead to enhanced Cd transport in rice [41]. Root anatomy plays a key role in Cd uptake and accumulation in rice roots.

Iron plaque on the root surface enhances plant tolerance to the environment, and Fe ion availability mitigates the toxicity of Cd to rice seedlings [42]. Compared to flooding, dry cultivation conditions [43] decreased DCB-Fe concentrations, which may be related to changes in the radial oxygen loss (ROL), Eh, and pH [44] in the rhizosphere soil. The DCB-Fe and root Cd concentrations were negatively correlated under Cd stress (Figure 8); this may potentially be due to Cd and Fe competing for the same transporters in the rice roots during absorption [45]. YJ37 had the highest concentration of DCB-Fe but a lower DCB-Cd concentration (Figure 7). The Fe and Cd concentrations in the root surface iron plaques significantly affected Cd uptake and accumulation in rice [46]. These iron plaques act as temporary storage for Cd [47] and reduce Cd uptake.

Dry rice cultivation has gained popularity and expanded its cultivated area due to its reduced labor requirements and water consumption. However, the impact of this cultivation method on Cd accumulation levels in grains remains uncertain. The higher Cd concentrations shown in dry-cultivation rice grains compared to flooded-cultivation rice [11] may pose greater health risks. Different rice varieties have different Cd bioaccumulation capacities. YJ37, as a low Cd-accumulating variety, should be screened to mitigate the increased health risks of dry-cultivation rice in areas with high Cd background values and combined with other measures to ensure safe rice production.

## 4. Materials and Methods

### 4.1. Pot Experiment

Five common rice varieties under dry cultivation conditions were selected from Yunnan Province, China. These varieties included DHY34, DHY615, DHY918, YY673, and YJ37. DHY34, DHY615, and DHY918 were supplied by the Centre for Rice Research of Yunnan Agricultural University, and YY673 and YJ37 were supplied by a seed company.

The soil was taken from the top layer of arable land at Yunnan Agricultural University (0–20 cm depth), naturally air-dried, and passed through a 2 mm sieve before impurities were removed. Next, a CdCl_2_·2.5H_2_O solution was added to prepare contaminated soils with different Cd concentrations (0, 10 mg kg^−1^), equilibrated for 15 days, and prepared for use. Before sowing, a basal fertilizer was applied (urea/phosphorus pentoxide/potassium oxide = 1:1:1 mass ratio), and the soil was watered to keep it moist [48]. The CK soil pH value, organic matter, total nitrogen, total potassium, total phosphorus, and total Cd concentration were 5.82, 5.79 g kg^−1^, 0.86 g kg^−1^, 5.28 g kg^−1^, 1.48 g kg^−1^, and 2.24 mg kg^−1^, respectively. The soils in the experimental plots were acidic because most agricultural soils are acidic in Southwestern China.

The rice seeds were disinfected using a 10% (*V*/*V*) hydrogen peroxide solution for 10 min, rinsed with deionized water, and placed into a Petri dish at a constant temperature of 25 °C until they germinated. Then, 8~10 seedlings were selected and sown in plastic pots containing soil for the test (each pot had an inner diameter of 23.5 cm and a height of 25.5 cm). To start the potting test, each plastic pot was filled with 5 kg of soil, and the rice was planted at the three-leaf stage, with five plants in each pot. During the three developmental periods, the soil moisture content was maintained at 60–70% of the field water-holding capacity using the weighing–watering method [49]. In this study, two Cd treatment concentrations, 5 rice varieties, 3 replications, and a total of 30 pots were used in a randomized block design.

The roots, shoots (three developmental periods), and grains (maturity stages) were harvested separately. To harvest the roots, the rhizosphere soil attached to the root surface was taken. The fresh roots were washed with tap water and deionized water and scanned using a digital root scanner before being soaked in a Na_2_-EDTA solution (20 mmol L^−1^) for 15 min to remove the Cd^2+^ adhering to the surface. The rhizosphere soil was naturally air-dried and passed through 0.149 mm and 2 mm aperture screens. The roots and shoots were rinsed clean with deionized water, swabbed dry, and then treated at 105 °C for 30 min before being dried at 70 °C to a constant weight and then ground and crushed for measurement.

### 4.2. Determination of Cd Concentration

The Cd concentration was determined in rice roots, shoots, and grains using 10 mL of HNO_3_-HCLO_4_ (1:9). The available Cd in the rhizosphere soil was determined by extracting a 5 g sample with 25 mL of DTPA. The soil Cd concentration was determined by extracting a 0.5 g sample with aqua regia/perchloric acid (15:3). The iron plaque was determined using the DCB method [50], and the concentrations of DCB-Fe and DCB-Cd were determined using flame atomic absorption spectrophotometry.

### 4.3. Determination of Rice Root Morphology and Observation of Root Anatomy

The fresh roots were scanned with a digital root scanner software (Epson Scan 2)) and quantitatively analyzed to determine the root length and root volume using the accompanying root analysis software, WinRHIZO 2013f. The fresh roots were cleaned, cut into 3–5 mm root segments, fixed in 70% FAA fixative, and sectioned using the frozen section technique. The sections were stained sequentially with Saffron O-Fast Green (Sigma-Aldritch, St. Louis, MO, USA), observed under a microscope, and photographed using a camera. The resulting images were analyzed to identify the exodermis, endodermis, stele diameter, and secondary xylem area [51] using image analysis software. (Image Viewer DPVIEW V2.0.10820).

### 4.4. Statistical Analyses

To study the distribution of Cd in the rice, the BCF and TF were calculated as follows:

BCF = Cgrain/Csoil(1)

where Cgrain and Csoil are the Cd concentrations in the grain and soil, respectively.

TF = Cshoot/Croot(2)

where Cshoot and Croot are the Cd concentrations in the shoot and root, respectively.

To study the effect of Cd stress treatment on the root morphology and anatomic structure of different genotypes of rice varieties, the rate of change was calculated as follows:

Rate of change = (C_CK_ − C_Cd treated_)/C_CK_ × 100% (3)

where C_CK_ is the value of the root length, root volume, exodermis thickness, endodermis thickness, stele diameter, and secondary xylem area under the CK treatment. C_Cd treated_ is the value of the root length, root volume, exodermis thickness, endodermis thickness, stele diameter, and secondary xylem area under the Cd stress treatment.

The arithmetic mean and standard deviation of three replicates were calculated. A one-way analysis of variance and a test of the significance of differences (Duncan, *p* < 0.05) were used to determine the differences among the five rice varieties. All statistical analyses were performed using IBM SPSS Statistics 21.0 for ANOVA, significance of difference tests, and correlation analyses (Pearson’s method, significance level *p* ≤ 0.05). Graphs were prepared using Origin 2021. SEM was applied to test for the direct and indirect effects of the Cd treatment, effective Cd, root surface iron plaques, root morphology, and anatomy on grain Cd concentration using Amos 26.0.

## 5. Conclusions

(1) There were genotypic differences in Cd accumulation in the different genotypes of rice studied, and the Cd concentration in each organ decreased in the order of root > shoot > grain. The different fertility stages showed that Cd accumulation decreased in the order of maturity >booting > tillering, and YJ37 was screened and determined to be a low-Cd-accumulating rice variety under dry cultivation conditions.

(2) Differences in the Cd uptake of rice were mainly induced by the available Cd concentration in the rhizosphere soil.

(3) Under the Cd stress treatment, the rice root length and root volume were inhibited, the root endodermis and exodermis were thickened, and the stele diameter and the secondary xylem area were reduced.

## Data Availability

Data are available upon request.
